# Resting heart rate as a predictor of metabolic dysfunctions in obese children and adolescents

**DOI:** 10.1186/1471-2431-12-5

**Published:** 2012-01-12

**Authors:** Ismael F Freitas Júnior, Paula A Monteiro, Loreana S Silveira, Suziane U Cayres, Bárbara M Antunes, Karolynne N Bastos, Jamile S Codogno, João Paulo J Sabino, Rômulo A Fernandes

**Affiliations:** 1 Department of Physical Education. UNESP Univ Estadual Paulista, Presidente Prudente, SP, Brazil; 2 Department of Physical Therapy. UNESP Univ Estadual Paulista, Presidente Prudente, SP, Brazil; 3 Department of Physical Education. UNESP Univ Estadual Paulista, Rio Claro, SP, Brazil; 4 Department of Physiology, School of Medicine of Ribeirão Preto, USP Univ of São Paulo, SP, Brazil

**Keywords:** Obesity, child, adolescent, metabolic dysfunctions, resting heart rate

## Abstract

**Background:**

Recent studies have identified that a higher resting heart rate (RHR) is associated with elevated blood pressure, independent of body fatness, age and ethnicity. However, it is still unclear whether RHR can also be applied as a screening for other risk factors, such as hyperglycemia and dyslipidemia. Thus, the purpose of the presented study was to analyze the association between RHR, lipid profile and fasting glucose in obese children and adolescents.

**Methods:**

The sample was composed of 180 obese children and adolescents, aged between 7-16 years. Whole-body and segmental body composition were estimated by Dual-energy X-ray absorptiometry. Resting heart rate (RHR) was measured by heart rate monitors. The fasting blood samples were analyzed for serum triglycerides, total cholesterol (TC), high-density lipoprotein cholesterol (HDL-C), low-density lipoprotein cholesterol (LDL-C), and glucose, using the colorimetric method.

**Results:**

Fasting glucose, TC, triglycerides, HDL-C, LDL-C and RHR were similar in both genders. The group of obese subjects with a higher RHR presented, at a lower age, higher triglycerides and TC. There was a significant relationship between RHR, triglycerides and TC. In the multivariate model, triglycerides and TC maintained a significant relationship with RHR independent of age, gender, general and trunk adiposity. The ROC curve indicated that RHR has a high potential for screening elevated total cholesterol and triglycerides as well as dyslipidemia.

**Conclusion:**

Elevated RHR has the potential to identify subjects at an increased risk of atherosclerosis development.

## Background

Over the last few decades obesity has reached epidemic proportions and become one of the major public health targets worldwide. Several researches indicate that obesity tracks from childhood to adulthood and constitutes a risk factor in the development of chronic diseases [1]. A high amount of body fatness is responsible for releasing a great amount of inflammatory adipokines into the bloodstream which has an important role in the pathogenesis of many chronic diseases [2,3], and also in the changes of sympathetic and parasympathetic activity in children and adolescents, which can result in an increased resting heart rate (RHR) [4-7].

In adults, the use of RHR as screening index for cardiovascular risk has been postulated [8,9] and supported by studies that reported its relationship to mortality, independent of abdominal obesity [10,11], but few studies are found which focus on the obese pediatric population.

Recently, Fernandes et al. [6] identified that a higher RHR was associated with elevated blood pressure, in both lean and obese male children and adolescents, independent of age and ethnicity, however, it is not clear if RHR can also be applied as a screening for other risk factors, such as hyperglycemia and dyslipidemia.

Thus, the purpose of the present study was to analyze the association between RHR, lipid profile and fasting glucose in obese children and adolescents.

## Methods

### Sample

One hundred and eighty obese children and adolescents (97 male and 83 female), aged between 7-16 years, from Presidente Prudente, western Sao Paulo State, Brazil, were analyzed. The subjects were invited, through television and newspaper advertising, to participate in an intervention program, with physical activity and nutritional orientation, for obese boys and girls (in the present study only the initial data was used).

The participants were contacted initially by phone, after which an appointment was made in order to take measurements at the Campus of the Universidade Estadual Paulista - UNESP. Primary obesity diagnosis was made using body mass index (BMI) according to the cutoffs proposed by Cole et al. [12]. After the preliminary diagnosis of obesity, the following inclusion criteria were used to select the subjects: i) aged between six and 17 years; ii) no engagement in regular physical activity within the three months prior to the study; iii) no limitations on physical activity diagnosed by a medical doctor; iv) a consent form signed by parents/guardians to participate in the study. The present research was approved by the Ethical Research Expert Committee of the Universidade Estadual Paulista - Campus of Presidente Prudente (protocol number 087/2008).

### Dual-energy X-ray absorptiometry (DEXA)

Whole-body and segmental body composition were estimated by Dual-energy X-ray absorptiometry (Lunar DPX-NT scanner [Lunar DPX-NT; General Electric Healthcare, Little Chalfont, Buckinghamshire, United Kingdom]) software version 4.7. Fat free mass (FFM), trunk fat mass (TFM) and percentage of body fatness (%BF) were measured. The DEXA and RHR measurements were made, on the same day, in a temperature-controlled room, in a laboratory at the University.

### Resting heart rate

Portable heart rate monitors (S810; Polar Electro, Kempele, Finland) were used to measure RHR (expressed as beats per minute [beats/min]), which was monitored during two 30-second periods (with a three minute interval between them) in the sitting position. All measurements were registered after five minutes at rest in a quiet room with a constantly controlled temperature [8]. For statistical analysis, values of RHR were stratified into quartile: Quartile 1 (< 72 beats per minute [bpm]), Quartile 2 (72 - 78.4 bpm), Quartile 3 (78.5 - 84.9 bpm) and Quartile 4 (≥85 bpm).

### Blood samples

Blood samples were collected with tubes containing EDTA and after a fasting night (10-12 hours). All collected blood samples (performed by nurses) and biochemical analyses were done in a private laboratory. The fasting blood samples were analyzed for serum triglycerides, total cholesterol (TC), high-density lipoprotein cholesterol (HDL-C), low-density lipoprotein cholesterol (LDL-C), and glucose, using the colorimetric method. Blood glucose ≥100 mg/dL was characterized as high blood glucose. Modifications in lipid profile were identified as: TC ≥170 mg/dL, LDL ≥130 mg/dL, HDL < 45 mg/dL or triglycerides ≥130 mg/dL [13]. The presence of, at least, one lipid modification was used to characterize the dyslipidemia diagnosis.

### Pubertal stage

The stage of puberty was self-assessed by the participants. The subjects received a standardized series of drawings to assess their own pubertal development. Girls received drawings of the five stages of Tanner breast and female pubic hair development with appropriate descriptions accompanying the drawings. Boys received drawings showing the five Tanner stages of genitalia and pubic hair development, with appropriate written descriptions [14,15]. The participants were asked to select the drawing of the stage that best indicated their own development (in cases where there was divergence between genitalia/breast stage and pubic hair stage [6% of the cases; n = 11], the genitalia/breast stage was adopted as pubertal stage). The results were placed by each subject in a locked box to guarantee the integrity and anonymity of the subjects, and only the main researcher had access to them.

### Statistical Analysis

Mean and standard deviation were used as central tendency and dispersion measures, respectively. Students' tests and one-way analysis of variance followed by a Tukey's multiple comparison test were used in the comparisons among independent groups. The Pearson product-moment correlation coefficient was used to analyze the association between RHR and biochemical variables. In a multivariate regression model, all biochemical variables with p ≤ 0.20 were simultaneously inserted, which should explain which biochemical variables could be used as a function of RHR (expressed as beta values [β]; adjusted by age, gender, %BF, pubertal stage and TFM). The receiver-operating characteristic (ROC) curve is a valuable tool for the assessment of the accuracy of diagnostic tests and provides a powerful means with which to assess the test's ability to discriminate between the true-positive ratio (sensitivity) and the true-negative ratio (specificity) [16]. For categorical analyses, the chi-square test (χ^2^) was used to determine the existence of a significant association between RHR quartiles and dyslipidemia. Statistical significance was set at < 5% and statistical software SPSS version 13.0 (SPSS Inc, Chicago, Illinois) was used for all analyses.

## Results

Table 1 shows the general characteristics of the sample stratified by sex. There was an average age of 11.2 ± 2.7 years, which was similar in both sexes. The males were taller, heavier and presented a higher amount of trunk fat. Comparisons of the %BF, between the sexes, were on the borderline of statistical significance. Fasting glucose (p = 0.064), TC (p = 0.640), triglycerides (p = 0.254), HDL-C (p = 0.271), LDL-C (p = 0.637) and RHR (p = 0.169) were similar in both sexes. Pubertal stages were similar in both boys and girls.

**Table 1:** General characteristics of obese children and adolescents (n = 180)

Variables	Overall sample (n = 180)	Male (n = 83)	Female (n = 97)	p*
	Mean ± SD	Mean ± SD	Mean ± SD	
Age(years)	11.2 ± 2.7	11.2 ± 2.6	11.1 ± 2.7	0.740
Height(cm)	150.1 ± 13.0	153.0 ± 13.8	149.1 ± 12.1	0.044
Weight(kg)	67.0 ± 19.2	71.9 ± 21.7	62.8 ± 21.7	0.001
FFM(kg)	33.5 ± 9.5	36.7 ± 10.8	30.7 ± 7.1	0.001
TFM(kg)	13.9 ± 5.0	14.8 ± 5.7	13.0 ± 4.2	0.017
%BF	45.7 ± 5.8	44.7 ± 5.6	46.2 ± 4.8	0.069
Pubertal Stages (%)				0.454^§^
I	36.1	36.1	36.1	
II	16.7	19.3	14.4	
III	21.7	24.1	19.6	
IV	16.7	12	20.6	
V	8.9	8.4	9.3	

*= Students' test for independent samples; § = chi-square test; SD = standard-deviation; FFM = Fat-free mass; TFM = Trunk Fat Mass; %BF = percentage of body fat.

Table 2 presents values of age and biochemical analysis distributed per quartile of RHR. The group of obese subjects with a higher RHR presented lower ages, higher triglycerides and TC. There was a similarity in trunk fat, fasting glucose, HDL-C, %BF and LDL-C.

**Table 2:** General characteristics of obese children and adolescents stratified by resting heart rate quartiles (n = 180)

	Resting	Rate	Heart	(beats/min)	
		
	Q_1 _(n = 44)	Q_2 _(n = 44)	Q_3 _(n = 45)	Q_4 _(n = 47)	
Variables	< 72	72-78.4	78.5-84.9	≥85	p*
	Mean ± SD	Mean ± SD	Mean ± SD	Mean ± SD	
Age(years)	12.4 ± 2.5^a^	10.9 ± 2.4	11.1 ± 2.6	10.2 ± 2.4	0.001
TFM (kg)	15,5 ± 5,1	13,4 ± 3,5	12,8 ± 5,5	13,5 ± 5,3	0.061
%BF	45.7 ± 5.8	46.1 ± 5.1	45.2 ± 4.3	44.9 ± 5.5	0.776
Glucose(mg/dL)	82.1 ± 5.5	81.8 ± 7.3	80.5 ± 5.9	82.3 ± 5.4	0.541
TG(mg/dL)	107.4 ± 40.9^a^	106.5 ± 51.1^a^	118.9 ± 48.1	140.9 ± 62.2	0.006
TC(mg/dL)	162.3 ± 32.4^a^	155.7 ± 30.9^a^	166.5 ± 28.1	178.3 ± 33.1	0.006
HDL-C(mg/dL)	43.4 ± 11.1	43.3 ± 10.1	43.1 ± 10.4	43.9 ± 9.3	0.978
LDL-C(mg/dL)	97.5 ± 29.3	91.1 ± 29.2	99.7 ± 24.3	106.2 ± 31.3	0.091

*= One-way analysis of variance;^a^= Tukey's test compared with Q_4 _(p < 5%); SD = standard-deviation; TFM = Trunk Fat Mass; %BF = percentage of body fat; TG = triglycerides; TC = total cholesterol; HDL = high density lipoprotein; LDL = low density lipoprotein.

A statistical relationship was observed between RHR and triglycerides and RHR and TC, but not between RHR and LDL-C (Table 3). In the multivariate model, only triglycerides and TC maintained a significant relationship with RHR, independent of age, pubertal stage, sex, general and trunk adiposity. There was a significant relationship between pubertal stage and TC (r = -0.15; p = 0.046), %BF (r = 0.19; p = 0.012), TFM (r = 0.60; p = 0.001) and RHR (r = -0.26; p = 0.001); but not for HDL-C, LDL-C, fasting glucose and triglycerides.

**Table 3:** Univariate and linear regression to describe the relationship between resting heart rate and metabolic variables in obese children and adolescents (n = 180)

Independent variables	Pearson's correlation		Linear regression	
	R	p	β*	p
Glucose(mg/dL)	-0.008	0.916	---	---
Triglycerides(mg/dL)	0.215	0.004	1.105	0.005
Total cholesterol(mg/dL)	0.189	0.011	0.613	0.014
HDL-C(mg/dL)	0.035	0.644	---	---
LDL-C(mg/dL)	0.118	0.115	0.327	0.148

*adjusted by gender, age, percentage of body fat, trunk fat and pubertal stages; SE = standard error; HDL-C = high density lipoprotein; LDL-C = low density lipoprotein.

**Figure 1:**
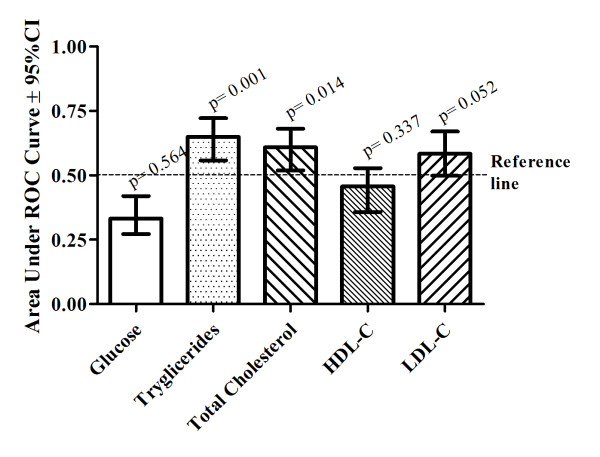
**Characteristics of resting heart rate to screen metabolic dysfunctions in obese children and adolescents**.

**Figure 2:**
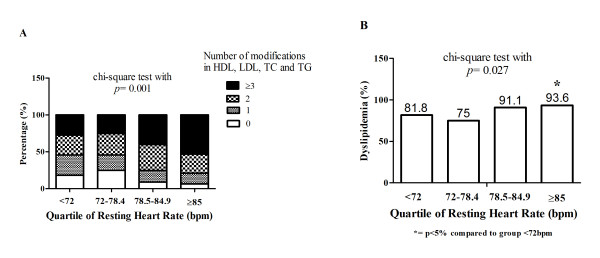
**Association between quartiles of resting heart rate and metabolic variables in obese children and adolescents**.

The ROC curve indicated that RHR has limited potential for screening elevated LDL-C (AUC: 0.584 ± 0.044; p = 0.052), but high potential for screening elevated total cholesterol (AUC: 0.609 ± 0.042; p = 0.014) and triglycerides (AUC: 0.650 ± 0.042; p = 0.001), as well as dyslipidemia (AUC: 0.658 ± 0.052; p = 0.010) (Figure 1). Finally, the number of modifications in lipid profile was inversely associated with RHR quartile (p = 0.001; Figure 2, Panel A) and the occurrence of dyslipidemia was higher in the higher quartile for resting HR (p = 0.027; Figure 2, Panel B).

## Discussion

The present study was carried out on obese children and adolescents, of both sexes, and identified that RHR has a significant relationship to dyslipidemia.

Previous studies have identified that, in children and adolescents, the chronological age is inversely related to RHR [6,17]. Al-Qurashi et al. [5] proposed age-specific reference values of RHR to Saudi children/adolescents, and identified that the RHR values were lower in adolescents than in children. A possible explanation for this is the alteration in the autonomic cardiac control, which is age dependent.

Previous studies carried out on subjects from birth to 24 years observed changes in the autonomic nervous system in accordance with nutritional status and advancing age [18,19]. They observed that sympathetic and parasympathetic activity increase in infants but, in children and adolescents, there is a great decrease in sympathetic activity and only a slight decrease in parasympathetic activity. Therefore, the lower cardiac sympathetic activity in children and adolescents may explain the reduction in the RHR values [5,6,17] observed in the present study, and support the necessity to adjust the statistical analyzes by age.

In this obese sample, both elevated occurrences of dyslipidemia (85.6%) and elevated blood pressure (17.8% [data not shown]) were observed. In fact, scientific literature has linked dyslipidemia and arterial hypertension to increased adiposity in children and adolescents [6,20,21]. In pediatric obesity, the endothelial dysfunction occurs due to a state of increased oxidative stress and the action of the vascular cells adhesion molecules [20,21]. Moreover, the above mentioned inflammatory mechanisms are strongly related to dyslipidemia [22]. Our data agrees with previous research, in which there is an elevated occurrence of the components of metabolic syndrome in obese Brazilian youths [23]. Caranti et al. [23] identified that metabolic syndrome has a higher occurrence in obese Brazilian youths (34.8% in boys and 15.6% in girls) than in obese Italian youths (23.6% in boys and 12.5% in girls). The above mentioned data reinforces the dramatic necessity to implement effective public health action, targeting the prevention of pediatric obesity in developing nations.

It is well established that the practice of regular physical activity improves the production of superoxide dismutase and nitric oxide [24,25] and, in turn, that regular physical activity from an early age, prevents the development of cardio-metabolic and cardiovascular diseases in adulthood [26]. In the present study, the sedentarism of the sample participants could be a factor in justifying the elevated occurrence of dyslipidemia and elevated blood pressure.

Research has shown an increased sympathetic activity in obese individuals [27-29]. Similarly, even in healthy normal weight subjects, the venous infusion of non-esterified fatty acids increases central sympathetic activation [30], while weight loss decreases sympathetic activity [31,32]. Our findings indicate the potential of RHR to screen dyslipidemia in obese children and adolescents. On the other hand, the observed relationship between tachycardia and dyslipidemia is not as simple to explain, because it is affected by many pathways and the causality in these biological mechanisms is still not clear [2].

The actual function of some adipokines that affect the insulin binding by blocking the insulin receptor substrates-1 activation, stimulate the lipolysis and contribute to development of dyslipidemia, was recently described [2]. These adipokines increase the production of reactive oxygen species in the brain, through activation of the nicotine adenine dinucleotide hydrogen phosphatase oxidase, increasing the oxidative stress in rostral ventrolateral medulla, which determinates the basal sympathetic activity [33,34]. In fact, recent studies have reported that the status of oxidative stress affects, positively, the sympathetic nervous system activation, which is responsible for the increase of RHR [33,34].

In our study, fasting glucose was not related to RHR. Oda and Kawai [10] identified, in a large sample of Japanese adults, increased fasting glucose in subjects with a higher RHR. Likewise, our results do not support these results, because our sample was composed exclusively of obese children and adolescents and further studies are necessary to clarify this issue.

A positive aspect of the present study is the analysis of TFM by DEXA. The inclusion of TFM in the multivariate model was important because this adipose tissue is related to the increased release of adipokines related to many pro-inflammatory mechanisms [2,3]. Moreover, our data indicated an important relationship between sexual maturity and higher TC, lower RHR and higher body fatness and, therefore, to take into account the pubertal stage in the analysis (instead of only chronological age) makes the findings more consistent, because sexual maturity is strongly related to factors that directly affect the RHR and lipid profile (e.g. hypertrophy/hyperplasia of adipose tissue, increased release of hormones and adipokines) [35].

On the other hand, some limitations must be pointed out. The cross-sectional design does not offer support to causality statements and, therefore, prospective studies from childhood to adolescence are necessary to describe more accurately the longitudinal relationship between RHR and dyslipidemia. The absence of inflammatory markers related to oxidative stress and, the absence of insulin measures to screen more clearly the relationship between RHR and glucose metabolism should be considered in future research.

## Conclusions

In summary, we conclude that increased RHR was significantly associated with dyslipidemia in obese children and adolescents and that elevated RHR offers potential to screen subjects at an increased risk of atherosclerosis development. However, longitudinal and epidemiological surveys should be carried out to develop optimal cutoff values for RHR in pediatric populations.

## Abbreviations

RHR: resting heart rate; BMI: body mass index; DEXA: dual-energy X-ray absortometry; FFM: fat free mass; TFM: trunk fat mass; %BF: body fat percentage; Beats/min: beats per minute; TC: total cholesterol; HDL-C: high-density lipoprotein cholesterol; LDL-C: low-density lipoprotein cholesterol; TC: total cholesterol; ROC: receiver operation characteristic; SD: standard-deviation.

## Competing interests

The authors declare that they have no competing interests.

## Contribution of the Authors

**RAF: **(1) conception and design of the study, (2) acquisition, analysis and interpretation of data, (3) draft of the article and selection of manuscripts to discuss the results, **PAM, LSS, SAU, BMA, KNB and JSC: **(1) Acquisition, analysis and interpretation of data, (2) draft of the article and selection of manuscripts to discuss the results, **IFFJ and JPJS: **(1) conception and design of the study (2) review and approval of the final version to be submitted. All authors read and approved the final manuscript.

## Pre-publication history

The pre-publication history for this paper can be accessed here:

http://www.biomedcentral.com/1471-2431/12/5/prepub
